# Linezolid Has Unique Immunomodulatory Effects in Post-Influenza Community Acquired MRSA Pneumonia

**DOI:** 10.1371/journal.pone.0114574

**Published:** 2015-01-30

**Authors:** Urvashi Bhan, Amy B. Podsiad, Melissa A. Kovach, Megan N. Ballinger, Venkateshwar Keshamouni, Theodore J. Standiford

**Affiliations:** 1 Division of Pulmonary and Critical Care Medicine, Department of Medicine, University of Michigan Medical Center, Ann Arbor, Michigan, United States of America; 2 Division of Pulmonary and Critical Care Medicine, Department of Medicine, Ohio State University, Columbus, Ohio, United States of America; Indiana University, UNITED STATES

## Abstract

**Introduction:**

Post influenza pneumonia is a leading cause of mortality and morbidity, with mortality rates approaching 60% when bacterial infections are secondary to multi-drug resistant (MDR) pathogens. *Staphylococcus aureus*, in particular community acquired MRSA (cMRSA), has emerged as a leading cause of post influenza pneumonia.

**Hypothesis:**

Linezolid (LZD) prevents acute lung injury in murine model of post influenza bacterial pneumonia

**Methods:**

Mice were infected with HINI strain of influenza and then challenged with cMRSA at day 7, treated with antibiotics (LZD or Vanco) or vehicle 6 hours post bacterial challenge and lungs and bronchoalveolar lavage fluid (BAL) harvested at 24 hours for bacterial clearance, inflammatory cell influx, cytokine/chemokine analysis and assessment of lung injury.

**Results:**

Mice treated with LZD or Vanco had lower bacterial burden in the lung and no systemic dissemination, as compared to the control (no antibiotic) group at 24 hours post bacterial challenge. As compared to animals receiving Vanco, LZD group had significantly lower numbers of neutrophils in the BAL (9×10^3^ vs. 2.3×10^4^, p < 0.01), which was associated with reduced levels of chemotactic chemokines and inflammatory cytokines KC, MIP-2, IFN-γ, TNF-α and IL-1β in the BAL. Interestingly, LZD treatment also protected mice from lung injury, as assessed by albumin concentration in the BAL post treatment with H1N1 and cMRSA when compared to vanco treatment. Moreover, treatment with LZD was associated with significantly lower levels of PVL toxin in lungs.

**Conclusion:**

Linezolid has unique immunomodulatory effects on host inflammatory response and lung injury in a murine model of post-viral cMRSA pneumonia.

## Introduction

Post influenza pneumonia is a leading cause of mortality and morbidity, with mortality rates approaching 60% when bacterial infections are secondary to multi-drug resistant (MDR) pathogens. MDR pathogens were initially only associated with hospital acquired infections. However, we have seen an explosion of community acquired pathogens that exhibit antibiotic resistance, infections which are associated with higher mortality and prolonged hospitalizations. One such pathogen is *Staphylococcus aureus*, in particular the emergence of community acquired MRSA (cMRSA) [1–3]. Severe necrotizing *S. aureus* pneumonia in previously healthy individuals (often with a preceding history of influenza-like illness) has been associated with the synergohymenotrophic exotoxin Panton-Valentine Leukocidin (PVL), and the genes encoding PVL are present in many cMRSA clones, including USA300 [4]. There exists literature to suggest an association of PVL with severe necrotizing infections; mechanisms by which this toxin induces tissue necrosis are not known. One possibility is that PVL-induced PMN lysis results in impaired host defenses, interfering with clearance of organisms from the site of infection and allowing unchecked bacterial growth and expression of other tissue-damaging exotoxins (e.g., α-hemolysin). Another possibility is that PVL itself directly or indirectly causes tissue injury [5]. In vitro, PVL activates PMNs to release potent pro-inflammatory mediators (IL-8 and leukotriene-B4) and granule enzymes (β-glucuronidase, hydrolase, and lysozyme) and to produce reactive oxygen metabolites that may cause tissue injury [6–10].

Linezolid (LZD) is an oxazolidinone, the first new class of antibiotic developed in the last three decades. Although this antibiotic is predominantly bacteriostatic, linezolid exhibits effective in-vitro and in-vivo activity against a wide variety of Gram-positive organisms, including methicillin susceptible *S. aureus* and methicillin-resistant *S. aureus* (MRSA) [11, 12]. Moreover, small studies have suggested that linezolid may be also effective in inhibiting PVL toxin, as well as other virulence factors observed in community acquired stains of MRSA.[13, 14]

The most common bacterial pathogens associated with flu pandemics are *Streptococcus pneumoniae*, *Haemophilus influenzae*, *Group A Streptococcus*, and *Staphylococcus aureus*. Staphylococcal pneumonia has been reported in previously healthy adults during influenza pandemics and epidemics for the last century [15–17], Mechanisms by which influenza may interact specifically with *S. aureus* include an influenza-induced increase in *S. aureus*–specific adhesion within the respiratory tract and expression of *S. aureus*–specific proteases, which may increase influenza viral replication [18–21]. This latter mechanism illustrates a reciprocal relationship in which *S. aureus* increases influenza disease severity while influenza promotes *S. aureus* infection and severity. Strains of influenza A virus also induce high IFN-γ levels as well as reduce phagocytic killing of *S. aureus*, leading to increased host susceptibility to bacterial superinfection [22–24]. No other respiratory virus appears to share with influenza such a prominent role in predisposing to and increasing the severity of *S. aureus* pneumonia [25].

Development of acute lung injury in patients with post influenza MRSA infections has been identified as one potential explanation for the excess mortality observed with this organism [26]. A meta-analysis of two studies performed by the same group of investigators using the same study protocol found that patients with nosocomial MRSA pneumonia treated with linezolid had a statistically greater survival compared to patients treated with vancomycin [27]. The enhanced ability of LZD, compared to vancomycin, to penetrate into lung tissue at therapeutic concentrations may explain the microbiologic and clinical differences observed in the available clinical trials [27–30]. Role of LZD in secondary bacterial pneumonia post influenza is still remains uncertain and needs to be investigated.

Recently, several classes of antimicrobial agents, including macrolides and quinolones, are reported to possess certain immunomodulatory effects [31–34]. In particular, protein synthesis-suppressing antibiotics, such as clindamycin and macrolides, can induce a general inhibition of virulence factor expression, such as alpha-toxin. Garcia et al. have recently shown that linezolid has potent concentration-dependent suppressive effects on cytokine production (TNF-α and IL-1) by LPS-stimulated monocytes *in vitro*, modifying the acute-phase inflammatory response by mitigating the cytokine cascade suggesting drug effects independent of its antimicrobial properties [35, 36].

In the present study we sought to evaluate the role of LZD in protecting against acute lung injury in post viral pneumonia caused by cMRSA. We found that even though there was no significant difference in lung CFU clearance and bacterial dissemination in mice co-infected with H1N1 and cMRSA, mice treated with LZD had decreased neutrophil accumulation and reduced inflammatory cytokine expression when compared to mice treated with Vanco. Moreover, LZD treated mice had significantly reduced lung injury as measured by protein leak, as compared to mice treated with Vanco, which was associated with significantly lower levels of PVL toxin in lung. These data suggest that LZD exerts advantageous immunomodulatory effects in a flu/MRSA dual infection model.

## Methods

### Mice

6- to 8-wk-old C57BL/6 mice were purchased from The Jackson Laboratory (Bar Harbor, ME). The animals were housed in specific pathogen-free conditions within the University of Michigan Animal Care Facility (Ann Arbor, MI) until the day of death. All animal experiments were performed in accordance with National Institutes of Health policies on the human care and use of laboratory animals and were approved by the University Committee on Use and Care of Animals (UCUCA) at the University of Michigan.

### Virus infection

A mouse-adapted influenza A virus strain (strain A/PR8/34: H1N1 isotype, ATCC) was inoculated into mice as described. Briefly, each mouse was inoculated intranasally with 50 µl influenza virus (10^3^ PFU/ml) per 20 g mouse. Inoculated viral titers corrected for mouse weight.

### MRSA inoculation

MRSA strains USA 300 obtained from Network of Antimicrobial Resistance in *Staphylococcus aureus* (NARSA), was grown in Nutrient broth (Difco, Detroit, MI) overnight at 37°C with constant shaking and quantitated by measuring the amount of absorbance at 600 nm and compared to a predetermined standard curve. Bacteria were diluted to the desired concentration for i.t. inoculation. Mice were anesthetized with ketamine and xylazine by the intraperitoneal route. The trachea was exposed, and 30 µl inoculum or saline administered via a sterile 26 gauge needle. An aliquot of the inoculated MRSA suspension was serially diluted onto nutrient agar plates to determine actual dose of i.t. injected bacteria.

### Antibiotic treatments

Starting 6 hours post inoculation, mice were treated with either LZD at a dosage of 80 mg/kg of body weight intraperitoneally, Vanco 110 mg/kg (intraperitoneally), or vehicle (control group). These doses were chosen based on studies that examined the pharmokinetic profile of these antibiotics in mice [37, 38].

### Bronchoalveolar lavage

At various times after i.t. inoculation, mice were euthanized in a rapid and painless fashion while deeply anesthetized. The trachea was cannulated with a plastic tube and the lungs lavaged with a 0.5 ml aliquot of calcium, magnesium-free PBS, or twenty 0.5 ml aliquots for collection of large numbers of AM. The cell-free BALF was collected, and the cells washed. In other instances, the cells were cultured for in-vitro studies. Lavaged cells from each group of animals were counted after lysis of RBC with hypotonic solution. Cytospins (Thermo Electron Corp. Waltham, MA) was performed for determination of BAL differentials using a modified Wright stain.

### Whole lung homogenization for CFU and cytokine analysis

At designated time-points, mice were euthanized by inhalation of CO_2_. The lungs were perfused with 1 ml PBS/5 mM EDTA and removed for analyses as previously described [39].

### Total lung leukocyte preparation

Lungs were removed from euthanized animals and leukocytes prepared as previously described [39]. Briefly, lungs will be minced with scissors to a fine slurry in 15 ml/lung digestion buffer (RPMI/5% fetal calf serum/1 mg/ml collagenase (Boehringer Mannheim Biochemical)/30 µg/ml DNAse (Sigma, St. Louis, MO). Lung slurries will be enzymatically digested, total lung cell suspension pelleted, resuspended and spun through a 20% Percoll gradient to enrich for leukocytes prior to further analyses. Cell counts and viability will be determined using trypan blue exclusion counting on a hemacytometer.

### Lung macrophage isolation

Lung macrophages (consisting of both alveolar and interstitial macrophages) were isolated from dispersed lung digest cells by adherence purification as previously described [40]. Cell preparations were generally enriched >98% for alveolar macrophage as measured by flowcytometry.

### Murine cytokine/chemokine and albumin measurement

(TNF-α, MIP-2, IFN-γ, IL-1β and KC; R&D Systems, Minneapolis, MN), and albumin (Albumin Quantification Kit; Bethyl Laboratories, Montgomery, TX) for lung permeability assessment were quantified using a modified double ligand method as described previously [41].

### Microscopic imaging

Lungs were fixed in 4% formaldehyde overnight, and 3-μm paraffin-embedded sections were stained with hematoxylin and eosin (H&E). A semiquantitative system was used to account for the degree of lung injury based on presence of inflammation, hyaline membranes and diffuse alveolar damage. A 5-point semiquantitative severity-based scoring system was used. The pathologic findings were graded as negative = 0, slight = 1, moderate = 2, high = 3 and severe = 4 in 10 non-coincident microscopic fields. A median score for each of the variables (0 = normal lung parenchyma; 1 = 0–25%; 2 = 25–50%; 3 = 50–75%; 4 = 75–100% of parenchymal structures altered) was then calculated.

### Western blotting

Lungs were harvested from infected and treated animals as described and homogenized to a single cell suspension. Cells were lysed in buffer containing RIPA (Sigma) supplemented with protease inhibitors (Roche Diagnostics). For immunoblot analysis, 20 μg protein was loaded onto 10% SDS-PAGE gels, subjected to electrophoresis, and transferred to membranes (Millipore). Membranes were incubated with Abs against PVL (1:1,000; Abcam) or β-actin (1:10,000; Abcam). Signals were developed with an ECL Plus Western blot detection kit (Amersham, Arlington Heights, IL).

### Methods of euthanasia

Anesthesia was induced by the inhalation of carbon dioxide. Following the induction of deep anesthesia, the animals were exsanguinated. This method is consistent with the recommendation of the Panel on Euthanasia of the American Veterinarian Medical Association.

### Statistical analyses

Measurements were determined for each individual mouse, and the means and SEM were generated for each treatment group. Differences between experimental groups were determined using t tests, one-way ANOVA, where appropriate. Differences were considered statistically significant when p < 0.05. Analyses were performed using GraphPad Prism software package (GraphPad Software, La Jolla, CA).

## Results

### Bacterial clearance and dissemination in LZD or Vanco treated mice in post viral bacterial pneumonia

**Figure 1:**
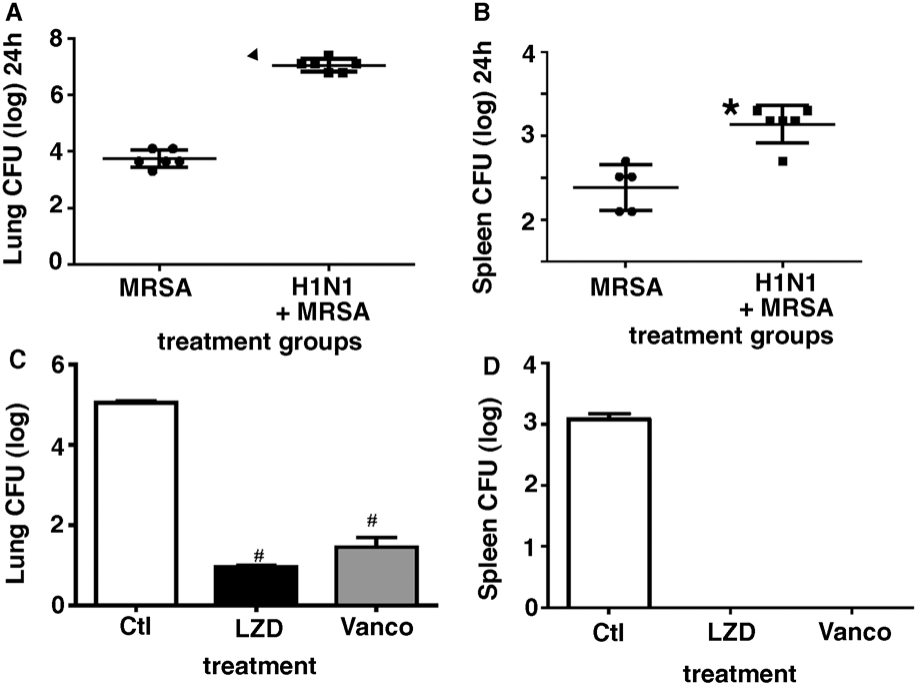
Mice infected with cMRSA post H1N1 have higher bacterial burden as compared to virus infected animals alone and treatment with antibiotics post bacterial challenge decreases bacterial burden in lung as well as protects against systemic dissemination as compared to saline treated animals. C57BL/6 mice were infected with 100pfu H1N1 i.n. and on day 7 challenged with vehicle or 5×10^7^ cMRSA, lungs and spleen harvested 24 hours post bacterial challenge and bacterial burden assessed (Fig. 1A, 1B). WT mice were infected with H1N1 100 pfu i.n. and on day 7 challenged with 5×10^7^ cfu cMRSA i.t., infected mice were treated with either vehicle or antibiotics LZD (80mg/kg i.p.) or Vanco (110 mg/kg i.p.). Lungs (Fig. 1C) and spleen (Fig. 1D) were harvested 24 hours post bacterial challenge and CFU quantitated. n = 6 in each group, experiments repeated thrice. #p < 0.01 as compared to vehicle treated group. Error bars represent mean±SEM.

To evaluate if bacterial clearance and dissemination post cMRSA infection would be altered by prior challenge with influenza, C57BL/6 mice were infected with 100 pfu H1N1 i.n. or saline, and on day 7 challenged with 5×10^7^ cfu cMRSA. Lungs and spleen harvested 24 hours post bacterial challenge and CFU assessed. Mice infected with H1N1 and then challenged with cMRSA had significantly higher bacterial burden in the lung, as compared to cMRSA infected mice alone (>1000 fold difference, p < 0.0001). Moreover, mice challenged with cMRSA post viral infection had significantly higher dissemination, measured by spleen CFU (>10 fold, p < 0.01) suggesting that prior influenza infection substantially impaired cMRSA lung bacterial clearance and enhanced systemic dissemination at 24 hours though by 48 hours mice were successful in clearing the bacteria and had no evidence of bacteremia (data not shown). LZD and Vanco are the two primary antibiotics used to treat MRSA pneumonia. To determine the role of these two antibiotics on lung bacterial burden and systemic dissemination in the dual infection model, WT mice were infected with H1N1 100 pfu (i.n.) and on day 7 challenged with cMRSA 5×10^7^ cfu (i.t.). Six hours post bacterial challenge mice were treated with either vehicle or LZD or Vanco i.p, lungs and spleen were harvested 24 hours post bacterial challenge and bacterial CFU determined (Fig. 1C). Mice treated with vehicle had high bacterial burden in the lungs and systemic dissemination as evident by high splenic CFU. By comparison, mice treated with either LZD or Vanco had a substantial decrease (>1000 fold, p < 0.01) in lung bacterial burden and no evidence of systemic dissemination, indicating that the two antibiotics were equally effective in decreasing bacterial burden in the lung at 24 hours post challenge.

### Treatment with LZD resulted in lower number of neutrophils in BAL, as compared to mice without antibiotic treatment or mice treated with Vanco

**Figure 2:**
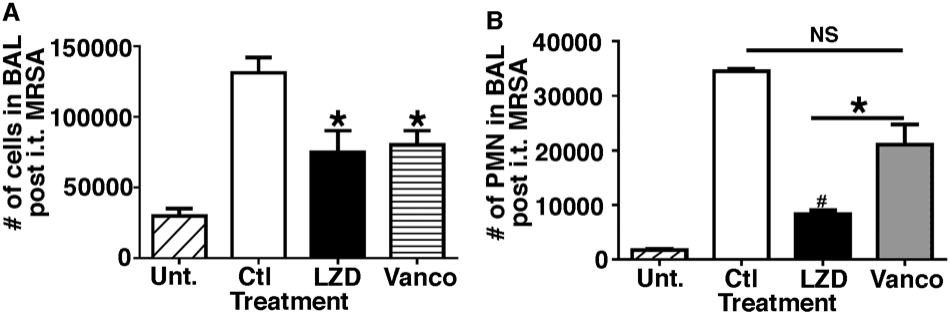
Mice treated with antibiotics have decreased total inflammatory cell influx as compared to saline treated animals; though LZD treated animals have lower number of neutrophils in BAL post bacterial challenge. WT mice were infected with H1N1 100 pfu i.n. and on day 7 challenged with 5×10^7^ cfu cMRSA i.t., infected mice were treated with either vehicle or antibiotics LZD (80mg/kg i.p.) or Vanco (110 mg/kg i.p.). BAL was performed 24 hours post bacterial challenge and total inflammatory cells quantitated using a hemocytometer (Fig. 2A) and neutrophils quantitated post cytospin (Fig. 2B). n = 6 in each group, experiments repeated thrice.*p < 0.05, #p < 0.01 as compared to vehicle treated group. Error bars represent mean±SEM.

Next we examined the effect of LZD or Vanco on inflammatory cell influx within the airspace in dual infected animals. Mice were sequentially infected with H1N1 100 pfu i.n., challenged with 5×10^7^ cfu cMRSA on Day 7, then six hours post bacterial challenge mice were treated with either vehicle, LZD or Vanco i.p. BAL was performed 24 hours post cMRSA administration, total cells were counted in each group and cytospins performed and stained with modified Wright’s stain to quantitate the total number of neutrophils. As shown in Fig. 2, mice treated with vehicle had significantly higher numbers of total inflammatory cell numbers and neutrophils, as compared to uninfected controls. The antibiotic treated groups had similar total cell numbers in BAL (Fig. 2A). However, mice treated with LZD had significantly lower number of neutrophils (9 ±0.6×10^3^, p < 0.01) as compared to mice that were treated with Vanco (2.3 ±0.3×10^4^) (Fig. 2B). This indicated that even though the two antibiotics were equally effective in eradicating infection, treatment with LZD resulted in an approximately 30% decrease in number of neutrophils recruited to the lung.

### Treatment with LZD resulted in decreased alveolar permeability, as compared to treatment with Vanco during post viral bacterial pneumonia

**Figure 3:**
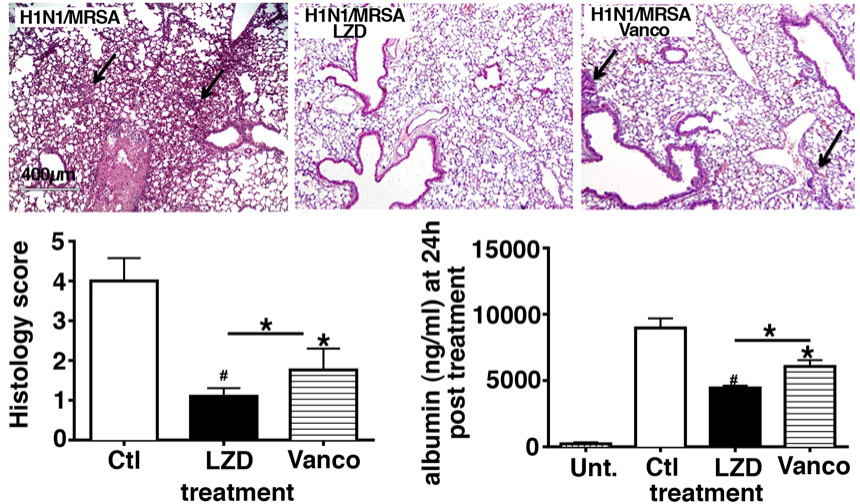
Mice treated with LZD have significantly lower lung injury as measured by protein leak as compared to Vanco or saline treated animals post bacterial challenge. WT mice were infected with H1N1 100 pfu i.n. and on day 7 challenged with 5×10^7^ cfu cMRSA i.t., infected mice were treated with either vehicle or antibiotics LZD (80mg/kg i.p.) or Vanco (110 mg/kg i.p.). Lungs were harvested 24 hours after bacterial challenge and fixed in formalin. Representative haematoxylin and eosin (H&E)-stained sections of vehicle and antibiotic treated animals 24 hours post bacterial challenge. Histology scores calculated. BAL was performed 24 hours post bacterial challenge and albumin measured by ELISA. n = 8 in each group, experiments repeated thrice. #p < 0.01, *p < 0.05 as compared to vehicle treated group. Error bars represent mean±SEM.

To determine if LZD differentially altered the course of acute lung injury, we assessed albumin leak as a measure of alveolar permeability in the murine model of post influenza pneumonia. Mice were sequentially challenged with virus and bacteria, treated with vehicle or antibiotics, then BAL performed at 24 hours and lung injury measured by quantification of protein leak (Fig. 3). Mice not treated with antibiotics had evidence of severe lung injury, as reflected by high albumin leak in the BAL fluid. By comparison, the antibiotic treated mice displayed substantially lower albumin in the BAL fluid. Interestingly, LZD treated group had significantly lower level of albumin as compared to the Vanco treated group (p < 0.01). Moreover, histopathology of lung sections from mice post infection and treatment with vehicle or antibiotics demonstrated increased evidence of diffuse alveolar damage, inflammatory cell infiltrate as well as fibrinous exudate in mice treated with vehicle (marked by arrows).Antibiotic treatment resulted in suppression of the inflammatory response and injury more so in the LZD treated group indicating that LZD reduced lung injury in the setting of viral-bacterial co-infection.

### Decreased levels of chemokines KC and MIP-2 and proinflammatory cytokine IFN-γ, TNFα and IL-1β in lungs post treatment with LZD as compared to Vanco

**Figure 4:**
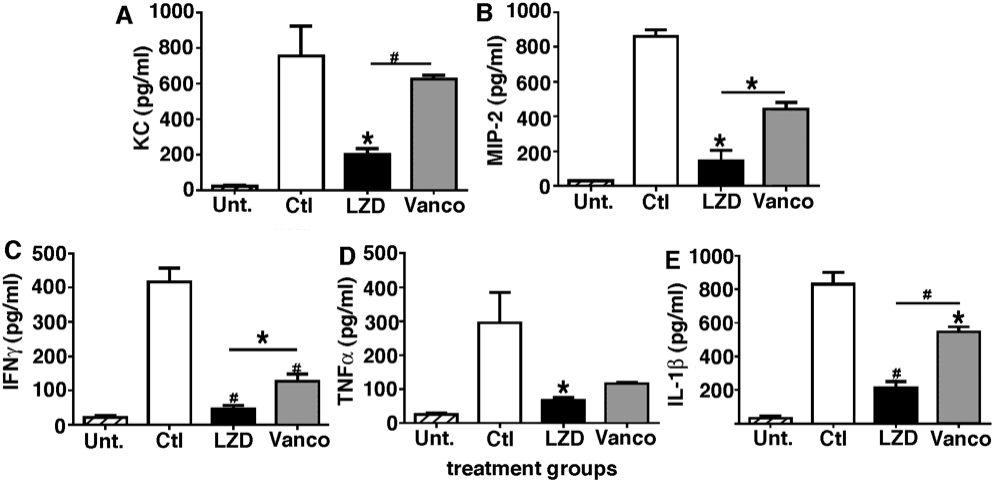
Mice treated with LZD have significantly lower chemotactic chemokines as well as inflammatory cytokines in BAL post bacterial challenge. WT mice were infected with H1N1 100 pfu i.n. and on day 7 challenged with 5×10^7^ cfu cMRSA i.t., infected mice were treated with either vehicle or antibiotics LZD (80mg/kg i.p.) or Vanco (110 mg/kg i.p.). BAL was performed 24 hours post bacterial challenge and chemokine/cytokine protein levels measured by ELISA.). n = 6 in each group, experiment repeated twice. *p < 0.05, #p < 0.01 as compared to vehicle treated group. Error bars represent mean±SEM.

Having observed significantly lower numbers of neutrophils and albumin levels in BAL of mice treated with LZD as compared to Vanco, we next assessed expression of chemokines in the lungs post bacterial challenge. Mice were infected with H1N1 100 pfu i.n. challenged with cMRSA on day 7, then treated with either vehicle, LZD or Vanco i.p. BAL was performed at 24 hours, and CXCLI/KC and CXCL2/MIP-2 were quantitated by ELISA (Fig. 4). Mice that did not receive any antibiotic treatment had higher KC and MIP-2 protein levels as measured by ELISA. In comparison, there was a significant reduction in KC and MIP-2 levels in LZD treated mice as compared to either animals receiving vehicle or Vanco (p < 0.05).

Patients with severe ARDS have elevated levels of TNF-α and IL-1β in BAL [42] recently Patel et al have shown that alveolar macrophage-derived TNF plays a crucial role in triggering alveolar epithelial dysfunction leading to non-cardiogenic pulmonary edema [43]. To further evaluate the mechanism by which LZD exerted a protective role in post viral bacterial pneumonia we harvested BAL from mice post challenge with virus and bacteria and treatment with either vehicle or antibiotics and measured levels of IFN-γ, TNFα and IL-1β by ELISA. As shown in Fig. 4, mice that did not receive any antibiotic had significantly higher levels of IFN-γ, TNFα and IL-1β. In comparison, there was a significant decrease in IFN-γ and IL-1 protein levels when mice were treated with either LZD or Vanco. Though there was a trend towards lower levels of TNF-α post treatment with Vanco, LZD treatment had significant blunting of TNF-α production by 55% as compared to control animals. Moreover, LZD treated animals had significantly lower levels of IFN-γ and IL-1β as compared to Vanco treated group, suggesting that LZD had unique immune modulatory effects on cytokine milieu.

### Alveolar macrophage phenotype post treatment with LZD or Vanco in mice with post viral bacterial pneumonia

**Figure 5:**
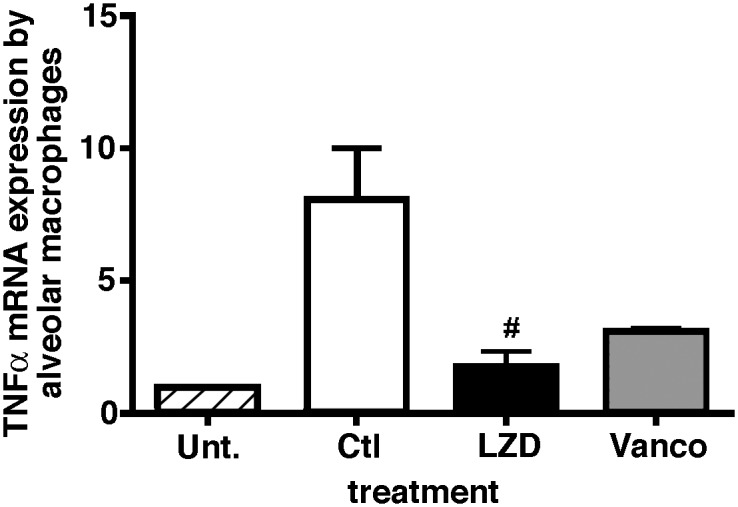
Macrophages harvested *ex-vivo* from LZD treated mice have decreased expression of TNF-α as compared to saline treated animals post bacterial challenge. WT mice were infected with H1N1 100 pfu i.n. and on day 7 challenged with 5×10^7^ cfu cMRSA i.t., infected mice were treated with either vehicle or antibiotics LZD (80mg/kg i.p.) or Vanco (110 mg/kg i.p.). BAL was performed 24 hours post bacterial challenge, macrophages harvested by adherence purification and TNF-α gene expression measured by real-time PCR. n = 5 in each group, experiment repeated twice. *p < 0.05 as compared to vehicle treated group.

Macrophages are the first line defense in lung antibacterial immune response, in part by ingestion and killing of microbes, and secretion of cytokines/chemokines to amplify the inflammatory response. We performed experiments to further evaluate macrophage function ex-vivo. Mice were challenged sequentially with virus and then bacteria, treated with either vehicle or antibiotics, then BAL performed at 24 hours. Alveolar macrophages were harvested by adherence purification and spontaneous expression of pro-inflammatory cytokine TNF-α expression measured by real time PCR. Alveolar macrophages from vehicle treated mice had robust expression of TNFα (Fig. 5). Even though mice treated with Vanco had a trend towards lower TNF-α expression, LZD treated mice had significantly lower expression of TNF-α, suggesting that treatment with LZD had an additional effect on macrophage function which could account for the protection from lung injury observed in this group.

### Mice treated with LZD have decreased level of PVL toxin in lungs as compared to Vanco treated group

**Figure 6:**
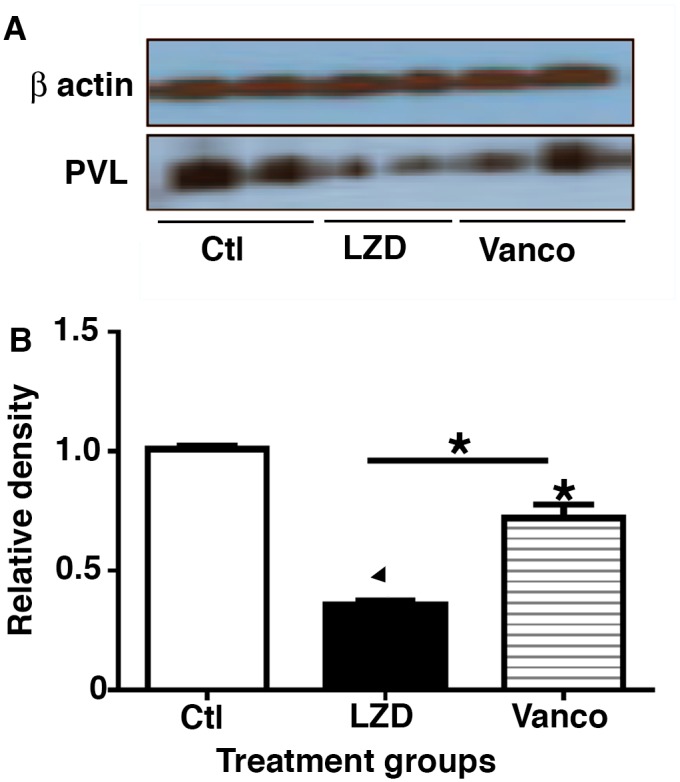
LZD decreased PVL toxin levels in lungs post bacterial challenge as compared to Vanco or saline treated animals. WT mice were infected with H1N1 100 pfu i.n. and on day 7 challenged with 5×10^7^ cfu cMRSA i.t., infected mice were treated with either vehicle or antibiotics LZD (80mg/kg i.p.) or Vanco (110 mg/kg i.p.). Lungs were harvested 24 hours post bacterial challenge and homogenized to single cell suspension, PVL protein levels were measured by western blot analysis and densitometry using Image J software. n = 4 in each group, experiments repeated twice. *p < 0.05, τ < 0.001 as compared to vehicle treated group.

Given that we found decreased neutrophilic recruitment, cytokine/chemokine expression and attenuated lung injury in mice treated with LZD post H1N1 and cMRSA infection, we evaluated comparative effects of LZD and Vanco on PVL toxin expression in the lung as a mechanism of immunomodulatory properties of the antibiotics. Mice were infected with H1N1 100 pfu i.n. and then challenged with cMRSA on day 7. Mice were treated with vehicle, LZD or Vanco i.p and lungs harvested 24 hours post bacterial challenge. Western blot analysis was performed to quantitate PVL toxin levels. As shown in Fig. 6 dual infected control mice had high levels of PVL toxin in whole lung, which was reduced by treatment with either Vanco or LZD. However, mice that were treated with LZD had significantly lower level of toxin present as compared to Vanco treated group (p < 0.05).

## Discussion

Acute lung injury/ARDS has been identified as a major cause of mortality and morbidity during pneumonia and ARDS in this setting is associated with worse prognosis. We found that LZD was as effective as Vanco in reducing bacterial burden in the lungs of mice infected with influenza followed by cMRSA, but decreased inflammatory cell influx when compared to mice treated with Vanco. This was associated with decreased production of chemotactic chemokines (KC/CXCL1 and MIP-2/CXCL2) and pro-inflammatory cytokine (TNF-α, IFNγ and IL-1β. Moreover, treatment with LZD protected mice from acute lung injury as measured by albumin in the BAL fluid. Importantly, the LZD treated group had attenuated level of PVL toxin in lungs post infection as compared to lungs harvested from Vanco treated animals.

Linezolid has been extensively studied in patients with MRSA pneumonia and has been proven to be superior to vancomycin for the treatment of MRSA nosocomial pneumonia. [12, 44] Considering the nature of recent Influenza epidemics and the morbidity associated with bacterial superinfection, we wanted to evaluate if antibiotic choice for treatment of secondary bacterial pneumonia specifically cMRSA would alter antibacterial host response.

Effective pulmonary host defense against respiratory pathogens is mainly mediated through phagocytosis and killing of microbes by alveolar macrophages and recruited neutrophils (28). Such defenses are orchestrated by a rapid inflammatory response after infection. Cytokines and chemokines are considered the engine responsible for coordinating this response (29). In particular, TNF-α and IL-1β share similar characteristics in promoting cell recruitment, activating respiratory burst, and increasing degranulation of macrophages [45](30, 31). However, these cytokines if expressed in uncontrolled fashion have been shown to be detrimental to the host and potentiate lung injury [46–48]. Moreover, mice lacking either IL-1 or TNF receptors have been shown to exhibit reduced lung inflammation in response to Influenza infection [49]. LZD has been shown previously to inhibit the synthesis of inflammatory cytokines in a concentration-dependent manner. A study by Garcia-Roca et al [35] demonstrated that release of IL-1β and TNF-α from lipopolysaccharide-activated monocytes was reduced after exposure to LZD. Similarly, a study by Takahashi and colleagues (34) reported that LZD inhibited the production of IFN-γ and TNF-α in lipopolysaccharide-stimulated whole blood. More recently, Breslow-Deckman have shown that LZD treatment decreases susceptibility to secondary bacterial pneumonia by decreasing IFN-γ levels in mice infected with influenza which is one of the first *in-vivo* studies demonstrating immunomodulatory effects of LZD and protection from secondary bacterial pneumonia though they did not use Vanco as a comparative agent or studied effects of LZD on lung injury [50]. In our study, we found reduced production of pro-inflammatory cytokines and chemokines *in-vivo*, as well as reduced expression of TNF-α mRNA from lung macrophages *ex-vivo* which was associated with reduced lung injury in the LZD treated group as compared to Vanco.

Alveolar epithelial cells are vital for the maintenance of lung function and the pulmonary air-blood barrier. In addition, respiratory epithelial cells respond to viral infections by mounting a cytokine/chemokine response that contributes both to the innate and adaptive host defences and have been shown to be stimulated by macrophages to secrete chemokines that can illicit an inflammatory response [51, 52]. We postulate that the decreased chemokine levels in whole lung post dual infection is likely secondary to attenuated epithelial cell damage resulting in decreased recruitment of neutrophils and protection from lung injury in mice treated with LZD [53].

The acute phase of ALI and ARDS is distinguished by the influx of protein-rich edema fluid into the air spaces as a consequence of increased permeability of the alveolar-capillary barrier. Lung edema, endothelial and epithelial injury are accompanied by an influx of neutrophils into the interstitium and broncheoalveolar space. Transmigration of neutrophils is a hallmark event in the progression of ALI and ARDS [54]. In patients with ARDS, the concentration of neutrophils in the bronchoalveolar lavage (BAL) fluid correlates with severity of ARDS and outcome [55–57], whereas the severity of lung injury has been reduced by neutrophil depletion in mice [58]. It has previously been shown that *S. aureus* and α-toxin from *S. aureus* can activate the NLRP3 inflammasome [59, 60]. Likewise PVL is a strong inducer of IL-1β secretion via a CTSB-mediated activation of the NLRP3 inflammasome [61]. In our study, we demonstrated suppressive effects of LZD on neutrophil recruitment, in association with immunomodulation of the lung cytokine milieu, which likely contributes to protection from acute lung injury which was measured by decreased protein leak in the lung.

Community-associated methicillin-resistant S. aureus, especially the pandemic USA300 clone, has been associated with severe infections and high mortality rates, particularly in patients with fulminant necrotizing pneumonia [62, 63]. USA300, like other cMRSA strains, produces Panton-Valentine leukocidin (PVL), a member of the family of bicomponent β-channel pore-forming toxins. PVL targets phagocytic leukocytes, especially polymorphonuclear leukocytes (PMNs). In vitro, PVL activates PMNs to release potent proinflammatory mediators (IL-8 and leukotriene-B4) and granule enzymes (β-glucuronidase, hydrolase, and lysozyme) and to produce reactive oxygen metabolites that may cause tissue injury [6–10]. PVL then lyse PMNs, resulting in the release of granule contents and reactive oxygen metabolites [7, 8, 64]. In turn, the toxic products derived from activated macrophages or lysed PMNs damage the alveolar epithelial and endothelial barriers, resulting in influx of fluid and protein from the vascular space into the airspace. Recently it has been shown that LZD suppress PVL production in patients with cMRSA [65]. Suppression of toxin production is a likely mechanism contributing to reduced pro inflammatory cytokine production and decreased lung injury observed in our model of post viral bacterial pneumonia.

In conclusion, using a mouse model of post viral bacterial pneumonia, our study suggests that even though there is no difference in bacterial clearance between the two antibiotics LZD and Vanco, LZD exerted unique immunomodulatory effects on toxin production and alveolar inflammation that may translate into improved clinical outcomes in patients with post viral bacterial pneumonia.
